# Health and environmental effects to wildlife from radio telemetry and tracking devices—state of the science and best management practices

**DOI:** 10.3389/fvets.2024.1283709

**Published:** 2024-03-06

**Authors:** Albert M. Manville, B. Blake Levitt, Henry C. Lai

**Affiliations:** ^1^Advanced Academic Program’s Environmental Sciences and Policy Division, School of Arts and Sciences, Johns Hopkins University, Washington, DC, United States; ^2^National Association of Science Writers, Berkeley, CA, United States; ^3^Department of Bioengineering, University of Washington, Seattle, WA, United States

**Keywords:** fish, wildlife, radio telemetry/tracking, RFID chips, PIT tags, data-loggers, radiofrequency radiation (RFR), electromagnetic fields (EMF)

## Abstract

This paper discusses the potential health risks and benefits to tagged wildlife from the use of radio tracking, radio telemetry, and related microchip and data-logger technologies used to study, monitor and track mostly wildlife in their native habitats. Domestic pets, especially canids, are briefly discussed as radio-tagging devices are also used on/in them. Radio tracking uses very high frequency (VHF), ultra-high frequency (UHF), and global positioning system (GPS) technologies, including via satellites where platform terminal transmitters (PTTs) are used, as well as geo-locating capabilities using satellites, radio-frequency identification (RFID) chips, and passive integrated responder (PIT) tags, among others. Such tracking technologies have resulted in cutting-edge findings worldwide that have served to protect and better understand the behaviors of myriad wildlife species. As a result, scientists, field researchers, technicians, fish and wildlife biologists and managers, plus wildlife and other veterinarian specialists, frequently opt for its use without fully understanding the ramifications to target species and their behaviors. These include negative physiological effects from electromagnetic fields (EMF) to which many nonhuman species are exquisitely sensitive, as well as direct placement/use-attachment impacts from radio collars, transmitters, and implants themselves. This paper provides pertinent studies, suggests best management practices, and compares technologies currently available to those considering and/or using such technologies. The primary focus is on the health and environmental risk/benefit decisions that should come into play, including ethical considerations, along with recommendations for more caution in the wildlife and veterinarian communities before such technologies are used in the first place.

## Introduction

Over the last several decades, much has been discussed in the global media, scientific community, and regulatory agencies about the effects to humans from nonionizing electromagnetic fields (EMF). This includes the extremely low frequency (ELF) bands used in powerlines and all electrical appliances, as well as the radiofrequency (RFR) bands used in popular wireless consumer devices like cell phones, wi-fi, and supporting infrastructure, among many other applications. There is now a large body of research that eventually led to the 2B “possible human carcinogen” classification assigned to ELF (1) and RFR (2) by the International Agency for Research on Cancer (IARC) at the World Health Organization (WHO), making EMFs comparable to the negative human impacts from lead, exhaust fumes, DDT, formaldehyde, and others.

However, what has not been carefully evaluated are effects from the same exposures to ELF-EMF/RFR from the use of radio-tracking technologies directly attached to, or in, marine and terrestrial wildlife by field researchers, environmentalists, government officials, veterinarians, and other well-meaning individuals. The use of such technology in both domestic pets/agricultural animals and wildlife populations is one aspect of the broader category of environmental radiation pollution, although it is rarely understood in that capacity. As a result of their use, not only are tagged species subject to both near-and-far field exposures, but both aquatic and terrestrial species that congregate, for example, in packs, herds, colonies, build nests, live in hives, or form migratory groups are collectively affected by cumulative exposures from near-and-far field sources. Most models of these tracking devices create functional nonionizing radiation exposures for tagged species and other nearby animals, as well as adding to low-level ambient environmental exposures, given the scale at which they are being deployed in wildlife species today. However, the effects are not being collectively evaluated for cumulative impacts.

The use of such devices has now become indiscriminate in favor of human research, curiosity, and entertainment. Given nonhuman species’ unique physiology and sensitive magnetoreception abilities—as evidenced by their evolutionary reliance on the Earth’s geomagnetic fields for a majority of their life-activities, including, for example, migration, mating, and food-finding—both natural and manmade EMFs can be highly biologically active exposures with the ability to affect nonhuman species at vanishingly low intensity levels (3–6). The radiation emitted from many—though not all—tagging-devices is relatively low. But since they are placed in extremely close proximity to body tissues with relatively high local tissue energy absorption, they can cause biological effects. It is quite possible that we are missing critical physiological effects across whole suites of wildlife species, as well as pets and other domestic animals, based on obsolete assumptions that low-level EMFs are simply too weak to adversely affect living tissues (7). Or we may be assuming that effects—if any—are so minimal that our curiosity about other species overrides their primary survival needs to simply be left alone. Potential exposures also include impacts from ELF transponders/cables used in aqueous environments that may create ecosystem-level effects to non-tagged species (4). With the continuing global growth of technologies that emit ELF-EMF/RFR, which are all biologically active exposures and can cause adverse effects to flora and fauna (3–6), this paper focuses on some of the key effects from radio-tagging and suggests some options to address them.

The use of such technologies in the U.S. dates back to initial studies in 1959 using VHF collars (8). Arguably the current state of fish and wildlife conservation/management, as well as our growing understanding of the needs of specific fish and wildlife populations, would not exist for many species without the use of radio tracking, data logger, and micro-chip devices. For example, tracking technologies helped us far better understand the deep dives, movement patterns, and home ranges of great white sharks (*Carcharodon carcharias*) (3); the record marine mammal migrations of humpback whales (*Megaptera novaengliae*) of up to 5,000 mi (8,047 km) from their winter breeding to summer feeding grounds (9); the annual “figure eight” migrations of up to 50,000 mi (80,470 km) of Arctic Terns (*Sterna paradisaea*) (3) and up to 40,000 mi (64,000 km) of Sooty Shearwaters (*Ardenna grisea*) (10); and the 2,000 mi (3,219 km) one-way and twice-yearly migrations of the Porcupine herd of barren-ground caribou (*Rangifer tarandus groenlandicus*)—the longest recorded annual migration of any land mammal (11), among many others. The knowledge gained from such devices not only aids human understanding but also our ability to protect and manage other species from human activities, as well as other factors that may be contributing to species declines and extinctions.

Over a 3-year period, one of the authors of this paper (12) captured 35 black bears (*Ursus americanus*) and radio-tagged, released and tracked 25 in Michigan’s northern Lower Peninsula, USA. He triangulated and tracked bears radio-tagged with 2 types of very high frequency (VHF) neoprene neck-attached collars (fixed and expandable) using hand-held and pole-mounted Yagi field antennas and VHF scanning receivers on the ground. He also located bears from fixed wing aircraft and helicopters—using wing strut and body-mounted Yagi antennas as well as scanning receivers—and found one bear had traveled 95 air miles (153 km) from its winter denning site to its summer range, returning the following fall. The goal of the project was to determine home ranges, movements, key concentration areas, and den site locations while investigating the impacts from humans on bears. However, at the time of the project, he was unaware—as are most wildlife biologists—of possible impacts from EMF on the tagged bruins.

Clearly findings from use of tracking technologies can be incredibly important, and in many cases critical for the protection, management, and recovery of species including those that are imperiled, such as Whooping Cranes (*Grus americana*) (13) impacted by collisions with transmission and distribution power lines (14). However, there are potentially serious downsides to using this technology. These must be recognized and ideally replaced with safer alternative technologies—where they exist—by field researchers, fish and wildlife biologists, wildlife veterinarians, and others.

### Nonionizing electromagnetic field pollution

The electromagnetic spectrum is divided into ionizing and nonionizing bands. The ionizing bands—such as X- and gamma-rays and cosmic rays—have enough energy to knock electrons off atoms and molecules, thereby affecting biological functions, such as DNA damage. Nonionizing EMF has traditionally been thought not to have enough energy to do that, with damage being limited to electric shock in the ELF-EMF range and tissue heating at high enough intensities in the RFR bands. But that does not mean that nonionizing radiation is incapable of a host of potentially deleterious biological effects below those thresholds, such as indirect DNA damage from free radical production, among many others (3–5, 7).

Because most radiotracking equipment—though not all (see below)—involves some form of energy transmission to reach distant receivers, and the increasing regularity with which it is used today in so many capacities in domestic animal and wildlife populations, a word about the Earth’s increasing ambient exposures is in order as these devices have now become a contributing factor to increased radiation exposure, among so many others.

In 2021, these authors published a 3-part series in the Reviews in Environmental Health (3–5) that mapped for the first time measured rising ambient levels of radiofrequency radiation (from 20 kHz to 300 GHz) in many global environments, including urban, suburban, and rural areas, and compared it to the increasing database of over 130 studies that found biological effects at vanishingly low intensities (equivalent to far field exposure intensities) in all taxa studied [see Supplemental material in Levitt et al. (3, 4)]. This data pairing, with over 1,000 citations, was clearly able to broaden today’s chronic low-level nonionizing radiation exposures to ecosystem levels.

Radiofrequency radiation is a form of energetic air pollution with contributors from all wireless devices and infrastructure today, including from personal and/or municipal wi-fi, cell phones, cell/broadcast towers, satellites transmitting globally including into wilderness areas, smart meters/appliances/homes, “personal” assistants, medical monitors, underwater cabling, and military uses to name but a few. But not all environmental conductive properties are the same, so exposures will greatly vary, and individual species have evolved very different electric and magnetic sensors accordingly. Aquatic environments, for instance, are a highly conductive medium with high attenuation more suited to ELF frequency electric and magnetic field effects, while air, which is less conductive with little impedance, is far more conducive to RFR transmission effects. Many aquatic species have evolved highly specialized sensory cells to detect and use very low levels of electric fields, whereas airborne avian species have developed acute perceptual abilities via different mechanisms in the eye and beak areas that can be stimulated by anthropogenic RFR (4) to the degree that orientation and migratory patterns can be altered. Different exposure parameters therefore would apply to specific environments, as well as potential adverse effects in tagged species from different frequency ranges. In other words, a GPS radio collar circling the neck of a bear—with transmissions concentrated in the head—in a forested region may have different effects on a female black bear and her nearby cubs than a RF transmitter imbedded in the body of a shark or whale, although effects could nevertheless be adverse to each for different reasons. The same principle would apply, for instance, to tagged avian species flying near transmission towers versus electrosensitive fish near underwater cables. (See below for discussion of known adverse effects.)

Such species-specific environmental study comparisons regarding radio-tagging have not yet been made, however. The use of the technology is far in advance of the nascent field of research regarding effects to tagged species let alone different environments. The bottom line is that the popularity of many radio-tagging devices is now among the contributors to ambient exposures in all environments, capable—at least in theory—of affecting different species in different environments very differently. Any number of complex variables go into the equation that are beyond the scope of this paper.

With the exception of some infrastructure towers and cell phones, most of the above-mentioned technologies, including radio-tagging equipment, are categorically excluded from licensing by government authorities because they transmit at RFR intensities <1,000 Watts. Most international exposure guidelines for humans are based on acute short-term high-intensity exposures that are capable of heating tissue in a 6 ft. (1.8 m) adult male. However, the research database, as shown in Levitt et al. (3–6) and Lai (7), verifies effects far below the current guidelines (see Discussion for further information on what human guidelines are based on). Radio-tagging is used today in species that range from ants to leviathans, not a 6 ft. (1.8 m) human model, and radio-tagging devices can entail a 24/7 exposure, depending on equipment and research project needs.

There are no exposure standards for nonhuman species for ELF-EMF/RFR (5) and post-radio-tagging attachment surveillance for health effects is all but nonexistent. Due to the fact that radio-tagging technology is directly placed on/in wildlife, often communicating at various times and sequences with distant receivers, these can be strong near-field chronic exposures certainly not recommended for humans. One can reasonably assume safety issues would apply for nonhuman species as well but this is an unregulated area where low-level transmitters are given the benefit of the doubt over potential biological reactions. When signals from such equipment remain stationary in a formerly mobile animal, it is assumed that the tracking device has timed-out and/or fallen off, or somehow been slipped off, when in fact it may have contributed either directly or indirectly to animal mortality from various causes—among them ELF-EMF/RFR exposure.

### Tracking equipment often used by wildlife biologists

With so many significant discoveries regarding animal species from various tracking gears already available to the scientific community, use of such devices will certainly continue. In fact, radio telemetry may provide the only source of crucial data, often at an affordable cost, where the variables inherent to wildlife are being observed. This includes studies on home ranges/territories, dispersal patterns/movement distances, flight orientations for birds/bats, breeding/wintering sites, feeding/roosting locations, resting/loafing sites, seasonal habitat uses, and migratory corridors, among many others. For instance, data from GPS collars have recently been used to protect critical wolverine (*Gulo gulo*) breeding habitats in Idaho, USA, from snowmobile disruption and noise (15)—data that otherwise might not have been available due to poor backcountry access, winter weather challenges, and monitoring difficulties for this rapidly-dispersing and highly elusive mustelid.

Below are examples of tracking gear and related transmitting equipment that comes in many varieties and sizes, with the understood objective in the research community of further miniaturizing transmitters, as well as reducing battery size and weight, including, where applicable, those with solar battery chargers.

1. *VHF devices:* Very high frequency (VHF) transmitters (between 148–174 MHz, and 215–235 MHz) are generally inexpensive, used in collar mounted, ear-tag attached, feather harness fastened, fin inserted, and surgically implanted device models that vary in size and weight for external use on everything from hummingbirds and bats, to elephants and big cats. They are also implanted in snakes, sea otters (*Enhydra lutris*), sharks, sea and terrestrial terrapins, tuna and other fish, and myriad other animals. Primary considerations for those attaching the devices are to maintain total equipment weight to no greater than 5% of the animal’s body weight (16), using lesser weights for birds and other animals. This old-school but still reliable VHF technology uses line-of-sight reception with a signal that is continuously transmitting and can be detected by a receiving device. Such signals, however, can be impeded by thick vegetation, weather, and terrain (12). Batteries can last for ≥3 years, depending on the models (12), with up to a projected 10 years for elk (*Cervus canadensis*) (17). Collar models with external antennas can extend their life by putting batteries into “sleep mode.” VHF gear may also include additional GPS (global positioning system) attached devices, which can add another frequency exposure. VHF devices are the most frequently used tagging devices. Due to the constant VHF transmissions, they create near-field exposures which are the strongest for the tagged species on which they are mounted or inserted.
2. *UHF devices:* Ultra-high frequency (UHF) transmitters (between 300 MHz and 3 GHz) and receivers are used to a much lesser extent than VHF devices, for example, to assess road crossings, den site locations, and use of water holes (18) where smaller antennas and shorter radio ranges are effective. While the wavelengths and antennas are shorter than those of VHF, the equipment is costlier, more specialized, and the wavelengths raise additional concerns over impacts from EMF as these frequencies at near-field intensities are equivalent to cell phones attached to animals, creating 24/7 exposures—something certainly not recommended for humans. UHF devices are also line-of-sight, plus its short wavelengths can penetrate obstacles, making it useful for specialized radio-collar studies on large wildlife in, for example, the jungles of Southeast Asia with thick vegetation where UHF transmitters and their antennas, in conjunction with VHF and GPS gear, may also be employed. GPS data may also be downloaded to a portable receiver through a programmed collar that transmits data at set intervals using a UHF modem (19).
3. *GPS systems*: Global positioning system (GPS) technology (in the L-band or 12 GHz frequency range) is used in tracking collars, which includes a GPS radio transceiver (transmits and receives) in the collar with the capability of picking up signals from sets of four special satellites. The computer in the receiver picks up, calculates and stores time and location data at set time intervals (e.g., every 6 h; shorter intervals use more battery power thereby reducing collar life). Those stored data can be retrieved once the collar drops off or the animal dies; can be transmitted periodically to sets of satellites for download to a researcher’s computer; or can be sent on a programmed schedule to researchers in the field or at a base station (19). Newer and more expensive Iridium collars use GPS and satellite telemetry, which are called platform terminal transmitters (PTTs), are useful for satellite tracking in remote and inaccessible areas. GPS systems that both transmit and receive signals therefore result in strong RFR exposure, often very close to the animal’s head. Collar weight and battery size will dictate a collar’s useful life. Other than the collar itself and possible accompanying attachment problems, such GPS collars may be safer from an EMF-exposure perspective since they transmit less often than most VHF/UHF devices. However, in order to reach distant satellites, GPS/satellite tracking devices require a much higher-powered transmitter attached to the subject animal, resulting in more RFR being released at higher power densities which increases near-field EMF exposures to the test animal, and also reduces the system’s useful life versus those of VHF systems. Clearly there are tradeoffs. Preferred GPS systems should not transmit often—if at all—as increased transmission power even at reduced transmission durations still subjects collared animals to EMF exposures. Preferred GPS systems can be used to accumulate data for later analysis when the computer is recovered. The equipment and satellite rental fees can also be expensive. Due to battery drain/life and satellite costs, GPS collars frequently only retrieve data at set intervals called the “duty cycle,” remaining inactive and in “sleep mode” during other periods. The strongest “peak” exposure occurs when the duty cycle fires up to reach a distant satellite. GPS is also used in bird tracking bands which collect tracking and monitoring location data. In some instances, these data can also be downloaded to a cellular communications tower or other receiving device. Any GPS system with up- and download capacity has a potential RF component but it may not be a constant signal. In other instances, the devices must be removed by researchers, requiring recapture of the tagged birds or bats with accompanying stress to the animals. Rule of thumb: the more expansive and complicated the tracking system, the more will be the cost both to the researcher and likely to the research subject due to more RFR being released.
4. *RFID:* Radio-frequency identification (RFID) chips are small tags—often the size of a grain of rice—that are attached or implanted under the skin—such as in our pets, especially dogs and cats—and in various species of fish, including the Pacific bluefin tuna (*Thunnus orientalis*) (20) where they may be inserted, for example, behind the dorsal fin. These tags do not require a power source, and contain encoded information which is read at close range by radio waves from a chip reader. Some chips have been so miniaturized that they can be attached to the backs of ants to track their movements and habitat choices (21). As a rule, RFID chips do not transmit RF continuously and only give up information when called for by the chip reader. But RFID chips have been known to migrate throughout an animal’s body to distant limbs and organs, sometimes causing infections. Certain cancers are also known to form around them (see below).
5. *PIT tags:* Passive integrated responder (PIT) tags, a variant of RFID chips, are used in a variety of wildlife research of small-scale insect and amphibian studies such as at wildlife crossings (22), and attached leg-bands or surgically implanted tags in songbirds (23), among others. Like RFID chips, PIT tags do not require a power source. They are glass-encased microchips that transmit a unique 10-digit alphanumeric identifier when they cross the electromagnetic field of a RFID antenna, usually ≤3 feet (1 m) from the antenna. When detected at an RFID antenna receiver location, the PIT tag code and detection time are recorded, allowing for the automated collection of large amounts of data. PIT tags only transmit when they cross an RFID antenna/chip reader. Although the antenna/chip reader is small, sometimes they are mounted on individual towers where space allows and where an antenna can be deployed, which then creates 24/7 exposures at that site to “read” both day-and nighttime migratory species data. Nature centers are erecting such networks, again without understanding the full exposure possibilities to workers, visitors, migratory, and local wildlife. (See Discussion for further information on one such system.)
6. *Geo-locators:* Light-activated geo-locators (also known as data-loggers or geo-loggers that are activated by visible light) are used to track migratory birds, which are generally captured using mist nets, then leg-banded, and fitted with a geo-locator using a lower back-pack harness that straps to the legs. The unit has a light sensor, internal clock, a tiny battery that can last >1 year, plus a miniature computer that stores light measurements for determining bird position using daylight readings. They can weigh only 0.3 g, allowing placement on birds weighing >7 g. The major disadvantage—other than the obstructive inconvenience of the entire harness attachment—is that birds must be recaptured, often at the same location where they were initially captured, resulting only in an overall 20% chance of recapture; all other loggers and target birds are lost to the research. In one study, tagged birds were recaptured 35% of the time compared to controls without loggers (24). This low recapture rate was attributed to effects from the weight of the loggers, adverse weather conditions, flights over large water bodies, and deaths. There is no way to estimate what contributing factor the loggers may have had on avian mortality, plus EMFs may have been a contributing but unstudied variable. Once birds are successfully recaptured, however, loggers are removed, and data downloaded and interpreted to assess bird movements. This tool provides important movement and migration information that otherwise would not be available (24, 25) but biologists are not sure at what cost to target birds. There may also be passive DC EMF exposures due to battery placement directly on avian bodies for extended periods of time and the artificiality of the harnesses may affect mate/breeding selection, movement and food finding abilities. Loggers may also be subtly affecting avian migratory perception abilities due to EMF exposure effects on the presence of magnetite and cryptochromes in avian eye and beak areas. For a full discussion of mechanisms of EMF interactions with animals, including effects on magnetite and crypto-chromes in birds, see references [3, 4].
7. *Hybrid radio-tagging systems:* There are new hybrid systems being created that combine many aspects of the above discussed radio-tagging devices with the purpose of predicting medical conditions that could affect domestic animals at a future date. The intention is to get animals proper veterinarian care in advance of conditions that can imperil an animal’s life if treated only after such conditions are revealed. Unfortunately, these hybrids appear not to be simple health recording/data gathering devices like human electrocardiograms or electroencephalograms, but something more EMF-interactive. Recent hybrids have been used on sport animals like race horses and racing dogs. These were primarily introduced in 2023 at commercial race tracks with much fanfare and produced interesting predictive results regarding future lameness from various causes based on manufacturer’s information. Unfortunately, greater-than-expected animal deaths were also seen at some race tracks where these devices were used. Only limited veterinarian reports have been released as of this writing regarding causes which included sudden lameness and death; therefore, much is still unknown. EMF was not considered to be a contributing factor but should be. Unfortunately, veterinarians know very little about bioelectromagnetics and it may not even occur to them that anthropogenic EMF—especially in the RF bands—is among the variables. What is described by hybrid manufacturers (26) indicates these may be new complex exposures, e.g., applied near-field pulses, which are more biologically active than non-pulsed fields (27), at 2,400 pulses-per-second on chest/abdominal/heart anatomical areas recording significant information from all regions of the animal’s body. These devices are being used during races when animals experience peak stress with significantly increased cardiac rates.

ELF and RFR are known to increase, reduce, and entrain heart rhythms in other animal models, including humans. In an unusual divergence from the research norm, human studies may tell veterinarians and wildlife researchers more about potential EMF effects in animals than our traditional toxicology research approach of using animal models to assist with human exposure regulation of EMF. For example, one recent paper—using high frequency EMF at 2400 MHz (comparable to wi-fi) and 2,600 MHz (comparable to 4G) for 5 min applied on the chests of 30 healthy young adult humans between 20 and 30 years of age—by Parizek et al. (28) found a shift in cardiac autonomic regulation toward sympathetic overactivity and parasympathetic underactivity indexed by heart rate variability (HRV) parameters during EMF exposure. They concluded that “…HF EMF exposure results in abnormal complex cardiac autonomic regulatory integrity which may be associated with higher risk of later cardiovascular complications…” The paper also contains a comprehensive reference list of other pertinent EMF/cardiovascular studies in similar frequency ranges. There is also a recent meta-analysis of ELF and RFR cardio effects that found patterns similar to the paper noted above although results were contradictory (29). The authors noted that the region of applied EMF may change the influence of EMF exposure on heart rate variability; that exposure of the head region to EMF may cause less variation in HRV while the chest may increase HRV. They added that EMF’s duration and exposure pattern (intermittent—e.g., pulsed—versus continuous) could be another critical factor causing different results.

Such effects could cause arrhythmia and sudden death but how this may translate to large body animals such as race horses under peak stress remains unknown. While not enough information has been provided by the manufacturers of tagging hybrids on horses—such as the multiple frequencies employed between the data gathered and GPS systems, or how the data tracker may possibly be communicating with internal RFID chips, dedicated data readers, and/or the use of cell towers to coordinate signal information at the track—the hybrids as currently designed are clearly subjecting animals to a novel energetic exposure with a sufficient database in EMF research to warrant caution if veterinarian/wildlife professionals chose to look into it. There is an immediate need to conduct cause-effect studies to determine if there is any relationship to such use and accompanying impacts—including EMF exposures—from these new hybrids. The benefit-of-the-doubt should not be given to the technology simply because current thinking presumes that such exposures are ‘safe.’

### Some possible alternatives to radio gear

There are promising non-invasive approaches and non-EMF technologies that can replace radio-tagging but they entail accompanying up- and- downsides. For example, some—like artificial intelligence (AI)—are in their infancy, while others are tried-and-true but involve labor-intensive field research techniques combined with the use of newer photographic equipment. Some non-invasive technologies may be available to individual researchers, but not to others due to cost and/or access (e.g., satellite imagery). And some tools may only have limited applicability to the research task at hand, including methods such as annual breeding bird surveys (30) and Christmas bird counts (31), both of which only provide trends regarding bird presence and population status at specific survey times and from specific locations. Although such information is general, trend data nevertheless continues to be critically important in assessing overall bird population status.

(1) *eBird and radar tracking:* An important trend-mapping effort is eBird, a community citizen-science platform operated by the Cornell Laboratory of Ornithology which was designed to assist bird watchers/enthusiasts, ornithologists, and migratory bird managers in tracking and cataloging bird sightings, ultimately resulting in a global database that allows the birding community to track bird locations and movements (32). It is an important tool for monitoring continent-wide bird migration in the U.S. Weather surveillance radars (WSR), using Doppler weather radar scans (between 24.05 and 24.65 GHz), along with mobile marine band radars (generally in the X-band 10.525 GHz and K-band 24.150 GHz frequencies) used by field researchers, can also provide key movement information, including chronology, especially during spring and fall bird migrations (33). At least 2 problems exist with the use of radars. First, radars generally cannot detect individual “targets,” but instead discern larger mass movements of many birds, bats or insects. Second, the problem with weather and marine radars dedicated for such use is that they create miles-wide high-power environmental ambient “screens” of RFR through which wildlife must traverse in order to be recorded. Radars also produce an independent, biologically active environmental exposure—far-field in radar’s case versus the near-field exposures of radio-tagging to individual species. Far-field exposures that strong and broad, however, may be capable of affecting bird, bat, and insect perception and throwing migratory behaviors off track. There is limited data on that in bat and some other species (4). To a tagged species, this constitutes a multi-frequency, near- and far-field exposure.
(2) *Satellite photography:* Satellite photography can be useful especially, for example, to discover new breeding colonies such as the endangered Emperor Penguin (*Aptenodytes forsteri*) colony recently discovered in the Antarctic (34). These images, however, may simply be too coarse-grained to provide precise colony-specific information, costs can be significant, and some of the satellite technology may not be available to the research community, especially if it is classified. However, this information may be the only source material for determining penguin presence and breeding sites which is critical knowledge due to the growing impacts from climate change as the ice melts and seas warm on that distant continent.
(3) *Camera traps*: Camera “traps” can also be useful and are a benign, non-invasive option for assessing wildlife presence (35). In some instances (e.g., Sumatran tigers [*Panthera tigris sondaica*], snow leopards [*P. uncia*], wolverines [*Gulo gulo*] and African wild dogs [*Lycaon pictus*]), they can identify individual animals based on unique pelage patterns and fur colors. However, camera trapping is labor intensive, site specific, cannot be used to estimate a population size or determine overall animal movements (other than at specific camera sites), plus high-quality cameras can be expensive.
(4) *Mark and recapture*: Mark-recapture techniques can be used to estimate local populations of animals, including what one of the authors of this paper, as previously discussed, utilized by passive ear-tagging all 35 of the captured black bears, with additional radio-tagging of a subset of 25 (12). The estimation techniques used to determine, for example, animal presence, density, size, and health in estimating and assessing the local population of black bears in a specific study area can be labor intensive, expensive, and may require minimum sample sizes, specific trapping gear, training and permits for immobilizing animals, among other issues (36). A related tool is the software package called DISTANCE that enables researchers to analyze distance sampling data to estimate density and abundance of a population (37).
(5) *Hair/scent/scat analysis:* Two additional non-invasive techniques are used in studies to determine species presence and/or absence, including minimum numbers present. These include the use of hairs gathered from target wild animals (e.g., grizzly bears [*Ursus arctos horribilis*])—collected at scent and marking stations via wire brushes—and later used for subsequent DNA analysis (19). The second involves scat analysis for DNA assessment and hormonal presence (38). Both are specialized and expensive techniques with limited uses (39).
(6) *Computer modeling/AI*: Computer modeling (e.g., knowledge gathered from eBird Status and Trends) (32) continues to be used to calculate specific avian and mammalian population and behavioral dynamics such as home range, territory, dispersal, and overwintering sites based on previously collected and current data, a significant portion of which was utilized from radio tracking datasets (33). With the rapid growth of artificial intelligence (AI), additional opportunities to model the dynamics of birds and mammals increase as the technology is refined. Critically endangered North Atlantic right whales (*Eubalaena glacialis*), for instance, are being visually identified by facial recognition (40), and Canadian scientists have developed an AI tool to better locate Atlantic right whales in Canadian waters (41). Scientists from Alberta, Canada, have used AI to develop an inexpensive non-invasive footprint identification technology to recognize individual black bear footprints—each, like our human fingerprint, is unique to individual bears (42). AI is also being used by the U.S. Fish and Wildlife Service to track and model the threatened Louisiana black bear population (43), while scientists at the University of Victoria, B.C., have developed an AI tool to facially recognize grizzly bears (44). As AI is refined, wildlife biologists suspect it may have the capability to calculate statistically significant home range size, average dispersal distance, primary seasonal feeding areas, and key den site locations for black, grizzly/brown, and polar bears (*Thalarctos maritimus*), among others, based on existing datasets. For any AI calculation/model, validation and ground-presence accuracy will be critical. For migratory birds, AI could be useful in calculating migratory corridors, overwintering and breeding sites, key nesting locations, and other variables, again based on existing data and validation. Such efforts need to be integrated with ongoing eBird and other programs (45).

The development of a tool similar to ChatGPT (46)—the new viral AI chatbot tool that continues to be controversial (47)—could be used in the future to program computer models for fish and wildlife, estimating the population variables just discussed above. As AI evolves, it may provide additional wildlife uses, reducing or even eliminating use of radio tracking gear and the negative impacts from its use on/in target animals. This, however, will require further study, assessment, and validation of AI as a wildlife tracking tool, integrated with other ongoing tracking computer models, before it can become a reliable substitute for use of radio telemetry. The use of AI in wildlife monitoring, assessment and management is still a nascent field.

### Biological and behavioral impacts from tracking gear

Most of the negative consequences from ELF-EMF/RFR have been documented in laboratory animals, domestic pets, and humans (3–7). Generally, the consequences and potential negative radiation impacts from using radio telemetry and radio tracking gear on/in animals in the wild are mostly undocumented but of growing concern (3, 48). Because of equipment availability, potential impacts to wildlife/pets/domestic animals are frequently dismissed or ignored in favor of potential human knowledge-to-be-gained, and field researchers may be quick to continue using tracking gear without understanding the full potential consequences of its use on/in birds, mammals, reptiles, amphibians, fish, or other targeted wildlife subjects. Contained within that professional bias, the risk/benefit assumption favoring assumed safety may be skewed against the fact that such exposures have been found to be a broad cellular stressor leading to many adverse effects (49), including in nonhuman species.

Below are some observed impacts from the use of tracking gear that include, but are by no means limited to:

- Benign growths/tumors resulted from external radio collar use (e.g., in American Kestrels [*Falco sparverius*]) (50).
- Tissue irritation, infection, and death from surgically implanted transmitters were seen in snakes (51) and otariids (52), among others. Where thermometry cannot be used, surgically implanted data loggers, and skin-attached PIT tags have resulted in irritation and infections in birds and mammals (23).
- Sarcomas and other malignant cancers have been seen in research animals and domestic pets (53–61) from implanted RFID chips, with some cases attributed to the chip casing materials but which may also be related to EMF functioning as an initiator and/or cofactor (62).
- Severe metabolic changes in animals exposed to 915 MHz RFID chips have been seen (63).
- Skin and internal irritations, sensitivity, and collar injuries are common—e.g., from tracking collars and/or skin-implanted gear that is too tight, not expandable, or insufficiently expandable as the animal grows—and may chafe and/or choke the animal as it attempts to remove the collar or other gear (19, 64). This was documented by one of the authors of this paper, who noted a study bear that had apparently strangled itself in its winter den, ostensibly attempting to remove the collar. He also noted neck chafing in several bears whose collars were too tight as the bears grew (12). At the time, breakaway neoprene collars were unavailable, except for expandable models with latex bands used on juveniles.
- Behavioral changes in tagged individuals (19, 64), as well as reduced breeding and/or survivorship (64, 65) have been observed.
- Increased vulnerabilities to predators (19, 66, 67) due to a weakened host and other species’ ability to perceive transmitted signals on/in tagged animals, cluing predators to their presence.
- Concerns continue over the duration of RFR transmitter attachment and migratory disruption in avian research subjects and different frequencies used (48, 68, 69).
- Micro-current exposures from batteries, antennas, computers, RFID chips, PIT tags, GPS collars transmitting to satellites, and other sources of RFR create additional and unmonitored independent exposures to wildlife and their habitats (3, 4).
- Reviews of negative effects from transmitting gear that can lead to data biases, as well as multiple confounding results, have been published. Effects included decreased animal productivity, changes in behavioral and movement patterns, increased energy expenditure, biased sex ratios, and reduced survival (48). However, EMF, thus far, has been largely excluded as a confounder (3, 4, 48) even when adverse effects have been found to be significantly associated with the duration of attachment of RFR transmitter devices to wildlife (48, 69).

From the observed effects noted above, caution is warranted. Before any tracking gear is deployed, each gear type should be assessed from the perspective of costs and benefits, both to impacts on individual target animals, as well as to the overall status of the fish or wildlife population being affected. Ethical issues must also be considered, and depending on the type of study, location, and responsible authority, approval of the gear used and study protocol by an ethics committee or panel may be required (39). If possible, post-tagging follow-up surveillance regarding the health status of tagged species should be conducted, with EMF exposures factored in. To date, EMF effects from use of radio telemetry and related tracking gear are poorly studied, and certainly poorly integrated with related areas of science, but they are potentially well understood if approached from a more comprehensive perspective. Post-tagging surveillance can assist with such integration.

### Modifying gear to reduce impacts to tagged animals

Over the past several decades, improvements and added safety features have evolved with wildlife radio tracking gear. These are designed to make the devices more specific to research needs, generally improve animal ethical and welfare issues, and usually make the devices safer for the target species. Some of these improvements may reduce EMF exposures, albeit unintentionally, at least in some instances. Examples of improvements, with reviews of their efficacy, include:

- Use of short-term break-away collars or tracking device features, for example, on marine mammals, sea turtles and seabirds (65).
- Timed bolt oxidation/breakage and collar release designed to occur over specific periods of time (e.g., months, a year, or longer) when placed on a target animal (19, 65, 70, 71).
- Timed deterioration and release of collar harness material (70–72).
- Expandable collars and harness materials that deteriorate (12, 19, 64, 65, 70–72).
- Exploding bolt collar release designs are considered a fast way to remove a collar from a target animal but may not be an “improvement” as this requires an additional built-in radio receiver and explosive charge transmitter with inherent risk to the animal from the explosion, stress, and enhanced EMF (19, 71–73). Technically it’s not supposed to kill/injure the target animal.
- Unfortunately, there are no viable options for eliminating EMF emitted from tracking gear altogether other than non-use, but reducing radiation emissions is possible by increasing the use of “sleep mode,” reducing the frequency of data transmissions over time, or changing to non-invasive options instead. Radiotracking by its nature involves RFR and other EMF exposures.

### Other considerations, special uses, and continuing concerns

In tracking mortality data, mortality monitors/sensors (19) can be mounted within VHF collar transmitter packages, usually inserted in dental acrylic or epoxy resin next to the transmitter, battery and external antenna lead. These sensors increase the beat/pulse rate of the signal when the collar becomes inactive for a specific programmed period of time and a microswitch activates the sensor. The increased beat frequency enables the researcher to target the transmitter, retrieving the lost collar and possibly determining, if applicable, the cause of death, or determining if the collar was simply dropped or pulled off by the research subject.

Another tool, the “immobilization or capture collar,” contains two imbedded syringes each with immobilizing drugs, a radio-administered firing mechanism, and the tracking radio collar with a transmitter. These, for example, have been used to locate and tranquilize gray wolves (*Canis lupus*) preying on or causing other problems with domestic cattle in Minnesota, USA. The researcher can then follow the radio signal to the collar of the immobilized subject depending on the duration of the drug’s immobilizing effect, and fire the second dart if necessary to continue immobilizing the subject (19).

Aside from the newest telemetry technologies with safety features such as timed break-away telemeter/collar options, lost collar signaling, and data-card download capabilities, there can still be difficulty removing such devices after attachment or insertion (3). For example, recapture may be difficult, with inherent concerns over drug sensitivity and overdose, and issues that accompany field surgery including infection, immobilizing drug interactions, and stress.

Collecting transmitting devices can be challenging, especially once an animal has died, or devices have slipped off and/or self-released in remote areas. In the study by one of the authors of this paper (12), one of his study black bears had been reported illegally killed. While driving through a town in Michigan, USA, he detected a very loud signal over his tracking receiver on the frequency of the missing bear—the strong signal coming from a neighborhood in town. Before a search warrant could be obtained to locate and search a specific house, the signal abruptly stopped, possibly because the suspect had seen the author and game warden, and the collar was subsequently destroyed.

Basic metabolic devices may be surgically implanted using RFID chips and PIT tags that can read such functions as internal body temperature, blood pressure, breathing rate, and metabolism, and transmit those data to a receiver or receiving antenna. This technology, however, is surgically invasive, the tags must be read by an electronic scanner, and the research needs can be very specific. Removing the tags requires surgery along with added accompanying complications from any surgical procedure, including untreatable infections after animals are re-released into the wild.

A general rule of thumb: as the number of devices is added in or onto a collar, the cost of the technology grows, the weight of the collar/transmitting device increases, the chances of negative behavioral impacts are elevated, and the amount of released EMF directly into test subject tissue swells. It is recommended that researchers install a tracking unit (i.e., radio collar, transmitter, antenna, and battery) total weight of no more than >5% of the animal’s total body weight/mass in attempts to minimize changes in its behavior, predator vulnerability, stress, and discomfort. For birds, that recommendation should not exceed 3% of body weight; for fish 2–3%; and for reptiles and amphibians 3–5% (16).

Among humans, wearing or carrying personal dosimetry devices has proven promising for capturing ambient cumulative EMF exposure data, particularly in urban areas (3). However, attaching such devices for the same purposes in remote areas to wildlife is ill-advised given the amount of tracking equipment already being used and the ongoing amounts of EMF already being released (3). Because moving animals are being subjected to varying fields of EMF as they traverse through their varying habitats, pinpointing EMF from a tracking device versus EMF from “electro-smog” in the field would be confounding, of little utility, and not worth the added risk to target animals.

There continue to be nagging questions about impacts from the use of radio tracking devices on tagged wildlife as well as the impacts of stress from marking, capture and recapture. Mech and Barber (19) make some specific suggestions to evaluate impacts. If, following recapture, the tagged animal maintains its weight, successfully mates, establishes or defends a territory, and otherwise appears to behave normally, project proponents could consider the effects of radio tagging to be minimal. This, of course, does not account for any long-term impacts (e.g., cancers and benign growths, and cumulative effects of EMF). However, if project proponents note signs of consistent weight loss from the first capture to subsequent recaptures (which infers hindered movements that make target animals more susceptible to predation, parasites, and disease), and/or chafing or hair loss under transmitting collars, mitigating steps need to be taken immediately. This could range from loosening a collar to removing the collar and releasing the target animal back into the wild after veterinary care is administered if warranted.

### Best management practices and best available technologies

The following section below is in no way intended to endorse radio-tagging/RFID insertion to wildlife in any form; it is merely to recognize the significance of such technologies to researchers in an effort to understand/protect wildlife and to suggest ways to minimize damage to target species from all the various factors explored in this paper. This includes reducing/minimizing EMF exposures which are a biologically active hidden variable in all environmental exposures, capable of affecting all species studied at low intensities (3–6). The authors do not endorse the use of such technology for entertainment purposes or by people ill-trained to use it (although we do recognize the significance of wildlife documentaries’ ability to inspire human awe and support of conservation measures). Nor do we endorse the erection of infrastructure—such as free-standing towers transmitting 24/7—that are required to capture data in remote areas to record/follow tagged migratory species or other wildlife. Such infrastructure is increasingly being adopted at nature and interpretive centers, as well as by government regulatory agencies like the U.S. Fish and Wildlife Service where one of the authors was a long-time researcher and administrator, without an understanding of RFR as an environmental genotoxin (7, 62, 74) to human and nonhuman species alike. (See Discussion for additional information on such systems.)

1. It is recommended that all options other than the use of radio telemetry be thoroughly evaluated—e.g., eBird, satellite imagery, computer modeling including DISTANCE sampling, mark-recapture, camera trapping, DNA analysis, AI, and others where applicable. As with any study, a detailed literature search and review are strongly recommended. This will help determine if a similar telemetry study on the same or closely related target species has already been conducted, with results published, and whether the results from a previous study make it unnecessary to conduct the proposed telemetry project. Where no alternative options and/or comparable studies exist, we recommend researchers subscribe to the following protocols summarized below:
2. As suggested by others (19, 39), we strongly recommend that any study involving wildlife radio telemetry undergo full peer and veterinary review prior to application for funding and project initiation. The review should include examination of the study objectives and methods, an evaluation of expected impacts and outcomes to target animals and their habitats, an assessment of adverse effects of the tagging method—including specific EMF exposures, (e.g., frequencies used, signal characteristics regarding pulse rates/peak exposures, and transmission power density)—how to ideally avoid or minimize those impacts, any permits required from permitting organizations and/or government entities, any required training for immobilizing and handling the target organisms, and an assessment of all ethical issues (16, 19, 39, 73).
3. Regarding any proposed study objectives, the following questions should be answered (or at least discussed) as part of the project, and should be included in the written section on objectives:

- Is the use of telemetry going to be important to conserving a wildlife population, including the tagged individuals affected?
- Is the purpose of the telemetry study for wildlife conservation?
- Will the capture, tagging, release and tracking of the target animals subscribe to all ethical standards and procedures applicable to handling of the specific wildlife species—including efforts to minimize stress and suffering?
- Can it be demonstrated that radio tagging is necessary to meet the proposed research objectives?
- Do the methods and benefits of radio tagging justify the project’s adverse impacts to subject animals and their target population, including from EMF exposure?
- Do the methods meet the precise objectives of the study, including data that will be required, duration of the study, proximity of radio-tagged subjects, and the proposed number of animals to be captured and radio tagged?
- Is the goal of the study to obtain tracking data which most closely reflect the natural behavior of the target species?
- Especially for capturing and transmitting birds, will the transmitter-to-body weight ratios (3% maximum) be closely followed (16, 39)?
- How do the project proponents intend to assess, raise awareness, and ideally mitigate for adverse effects from use of the tracking gear on patterns of animal behavior for the target species, its survival, and reproductive success? Has EMF been factored in as a potential variable?

4. Any researchers planning a radio-telemetry study should strive to ensure that all study animals are affected as little as possible by the transmitter and antenna, and that the study animals are handled humanely and professionally during capture and transmitter attachment. Wildlife capture techniques should be designed to minimize stress to the target animals at all times, based on an understanding of the behavioral and physical characteristics of the target species (16, 19, 39, 73, 75, 76). Only experienced and ideally well-trained fish and/or wildlife professionals proficient in fish and/or wildlife capture, immobilization and handling should carry out the actual marking and tagging operations (39).
5. Experienced, ideally published researchers who have conducted similar telemetry studies should be contacted by the project proponents. These researchers can provide valuable information and guidance regarding transmitter size, weight and designs specific to the target species, attachment methods and capture protocols—including what worked and what did not—and can help to avoid problems that have already been solved by other professionals (16, 19, 39). To minimize adverse effects to the target animals, use radio tracking methods and select devices already used and studied, preferably on the same species. Tags should be of a minimum size, weight and configuration appropriate to the target species, its behavior and its habitat (16, 19, 39, 73, 75).
6. Current trapping and handling guidelines should be carefully reviewed and followed. These include use of the best and safest types of traps used to live-trap target animals, including use of trap transmitters on large carnivore traps to minimize trap-related injuries and capture-related stress, use of the best and safest immobilizing drugs, recommended handling procedures including how to deal with any overdose, shock and injury issues, seasonal dosing differences, and suggested collar tightness on all subjects (16, 19, 39, 73, 75, 76).
7. Specific recommendations include:

A. Use the lightest-weight transmitter package possible with a non-restrictive harness and an antenna that will not snag on vegetation, impede movement and increase target species vulnerability to predation. If possible, place and/or direct antennas away from the head.
B. Select an inconspicuous package, especially when dealing with animals that rely on cryptic coloration.
C. First test the transmitter packages on captive animals in a variety of environmental settings. This is important where marking procedures to be used are new to the target species, or new to the specific population of that species.
D. To minimize research biases, wait 1 week after target animal release before collecting data for analysis, thereby allowing for target animal’s adaptation to the device.
E. Avoid handling and instrumenting any target animals during any critical life history period, especially during reproduction and infant/chick rearing.
F. Radio tagging should not compromise the conservation needs and recovery goals for state and/or federally listed threatened and endangered species.
G. To minimize stress during handling, be as gentle as possible and keep handling to the shortest duration practical.
H. Treat any injury that resulted from marking target animals, and if an injury is serious, the animal should be euthanized.
I. Avoid transmitting parasites and infectious diseases between target animals during the marking procedures (77, 78).
J. Note that surgically implanting transmitters, whether in the field or at a clinic, involves trauma to the target animals, and may require recapture to administer follow-up care. Avoid these as much as possible.
K. Whenever possible, choose non-invasive tagging designs that can be kept in sleep mode when data/information are not being called for.
L. Whenever possible, monitor the health and welfare of all marked animals, including any discernible negative effects from EMF which can appear as burns, skin lesions, malaise, malnourishment, behavioral changes, breeding insufficiency, confusion and disorientation (3–6, 12, 19, 39, 73, 75).
M. Know the electromagnetic frequencies of the gear being used—often listed on manufacturer’s packaging or on the Internet—and search the bioelectromagnetics databases on PubMed and elsewhere for studies that may impact target or similar species. Become familiar with the cross-disciplinary nature of the subject.
N. In designing all studies, where possible, incorporate the environmental mitigation techniques of the “3-R’s”—reduce, reuse, and recycle.—*Reduce* the use of tracking gear as much as possible, e.g., do not tag 50 target animals when the same data can be gathered with 10–20; reduce the frequency of animals used, e.g., do not use the same animals repeatedly, especially endangered or species of special concern; and reduce study timeframes and EMF exposures to As Low As Reasonably Achievable (ALARA).
-*Reuse* all pertinent published information before commencing a new study as a derivative of personal ambition, curiosity, and/or desire; reuse gear whenever possible after it is cleaned and sterilized to reduce infection risk.
-*Recycle:* Air, water, and the electromagnetic spectrum are finite resources. Today’s radio-tagging technology employs a known genotoxin (7, 27, 49, 74) attached on/in sensitive nonhuman species. Every piece of radio tracking gear *not* used is like a plastic bag taken out of the waste stream.

## Discussion and conclusion

The use of RFR radio-tracking devices of all varieties has increased incrementally as the technology has evolved and continues to do so (79). Since VHF collars were first used in 1959 in research (8), many new designs, types, applications, combinations and technologies have been adapted to wildlife study, agricultural animals, and domestic pets. (80). Today it would be difficult to find any area of wildlife investigation that does not use radiotelemetry in one form or another. But because the total in-field use of most telemetry models is considered proprietary by the manufacturers who do not publish that information, it is not possible to provide comprehensive data on the cumulative numbers or types of gear in the field, although the types of radio-tracking devices most often deployed by wildlife professionals are summarized above. No one has tried to investigate just how much gear is in use today and/or if there are concentrations in certain research sectors. And there is a lack of numerical data on dosimetry of the field emitted and energy absorption in the higher frequency ranges because basically no research has been carried out on those aspects.

Other than Mech and Barber (19), there are few wildlife biologists who have published in the wildlife and conservation literature about possible RFR impacts of radio telemetry equipment on wildlife, acknowledging that there could be ill effects to target animals. However, in their published 2002 critique of wildlife tracking in national parks, they concluded that the radiation from transmitters was so low that ill effects seemed unlikely. While they also acknowledged that the radiated power from satellite platform terminal transmitters (PTTs—mentioned above under “GPS Systems”) was several orders of magnitude greater than those of conventional animal tracking transmitters (e.g., VHF radio collars, ground-based GPS collars, and data-loggers), no findings of detrimental effects to animals during these 360 mS transmissions from PTTs had been noted.

More recently, it has been documented by Balmori (48), and Levitt et al. in 2021 (3–6), that very low intensities of radiation barely above natural background levels can, and are, causing ill effects (e.g., DNA strand breaks, cancers and benign growths, metabolic changes, animal disorientation, fertility problems, and behavioral abnormalities, among many others) in laboratory test animals, domestic livestock, and wildlife too (3–6, 48, 69) across all taxa studied. There is a decades-long wealth of information on EMF effects on animal species in the bioelectromagnetics literature based on nearly 100 years of research on animal models intended to determine human safety parameters to EMF exposures. Bioelectromagnetics, however, is a discipline where wildlife biologists and environmentalists rarely read as it is related to the non-living disciplines of physics and engineering with its own nomenclature, measurement protocols, and standards of “proof.” Often the so-called “hard sciences” of physics and engineering are not only foreign to biologists, but can also be intimidating. This entire area suffers from over-specialization and deep siloes within the major branches of science at a time when cross-disciplinary integrative approaches have become crucial due to the increasing global tech-loving human population and escalating layers of new radiating technologies. EMFs in all frequencies are highly biologically active cumulative exposures. RFR is now a form of ambient energetic air pollution (3–6) given the scale at which it is deployed into the environment today.

It is this new understanding of low-level EMF effects to all species that calls into question the here-to-fore widespread assumption that radio-tagging is a benign activity below certain regulatory tissue heating thresholds (81–83). This is an assumption that has proven inadequate to the task of regulating for chronic low-level exposures (3, 84); unusual signaling characteristics that are not taken into consideration in any standards set for human exposures let alone for nonhuman species (27); and the fact that manmade EMFs are fundamentally physically different than anything that exists in nature (85, 86) to which nonhuman species are uniquely ill-adapted (3–6, 87–92). Human curiosity—when all these flaws regarding popular assumptions are factored in—does not supersede potential effects to animals but it does illustrate that new perspectives and research are needed.

The bottom line: very little EMF follow-up data have been collected within the wildlife biology community involved with the majority of radio-tagging activities, in part because such data were deemed outside their purview and/or unnecessary. Consequently, relatively little has been published in wildlife or conservancy journals on the subject and any that does exist typically comes from the bioelectromagnetics community, contained in related journals on EMF. But some information on radio-tagging is now being published in environmental journals, including works on possible ill effects to fish and wildlife from the use of radio collars and other tracking devices (3, 48, 93).

What cumulative contribution, if any, such devices may be adding to ambient EMF exposures at ecosystem levels in affected wildlife habitats from the gear’s artificially introduced exposures in formerly pristine areas has not been studied, nor are the impacts of telemeters in or on wildlife (3) in general despite research that could apply if we chose to do so. It would be helpful if the wildlife community—since they are the ones in the field—became more proficient in the recent literature contained within other disciplines on the subject, as well as more alert to potential adverse effects in targeted research species from such exposures. Field researchers may be witnessing but not knowing how to interpret such effects, or attributing them to unrelated things. EMF is an important independent research variable as both initiator and co-factor exposures (62). At present, there is unfortunately little coordination between wildlife biologists using such gear—and therefore in the best position to study EMF effects in tagged species—and scientists in the bioelectromagnetics community who know how to interpret such data which often include nonlinear effects with the greatest impacts seen at lower intensities (94).

A classic example of this disconnect is the growing installation of a collaborative international/national/regional geo-tracking network called “Motus” (Latin for “movement”) which tracks and coordinates the migration of birds, bats, some insect species, and some ground animals that have been fitted with geotagging transmitters that send a pulsed RF “ping” several times per minute, 24/7 for variable periods. The tiny batteries in these devices last from 20 days to extended periods, depending on the models used, and the devices weigh from 0.2 to ~2.6 g. The signals are uniquely digitally encoded to the tagged subject with information collected by special antennas as various tagged species fly or walk by. The information is stored for future transmission to researchers on predetermined time lines. The tags have a power density strong enough to reach the receiving antenna up to 12 miles (19.3 km) away (95). Motus uses a battery-powered radio-tag with an attached long antenna that can exceed the length of the targeted species. The apparatus is attached to a harness that loops around the bird’s chest and legs. Transmitters attached to insects, such as butterflies, are often embedded.

With Motus networks, tracking tags come in 2 models: Lotek and Cellular Tracking Technology (CTT) (96). For detecting Lotek tags, antennas must be tuned to the frequencies 150.1 MHz, 151.5 MHz, or 166.380 MHz, depending on the location of the tracking effort being conducted globally. For CTT tags, antennas must be tuned to 434 MHz.

Because migratory species do not recognize national borders, the benefit of a collaborative international migratory species tracking system is obvious for future identification of threatened species and concomitant conservation efforts. Motus has become a popular public/private partnership between the U.S. Fish and Wildlife Service (USFWS) (95) acting in an advisory capacity with national wildlife conservation sites and nonprofit nature conservancies. Unfortunately, the Motus buildout, in the opinion of these authors, has largely been done without significant evaluation by the USFWS, or other vested agencies like the U.S. Environmental Protection Agency, and the Federal Communications Commission regarding deployment, training, and radiation effects. There is no government oversight once systems have been built and are operating, and enforcement generally occurs only when gross violations are voluntarily reported regarding certain protected species (5). No National Environmental Policy Act (NEPA) environmental assessments (EA’s) or environmental impact statement (EIS’s) studies have been conducted on Motus. There has been no discussion of the potential ELF-EMF/RFR biological effects to tagged species, or the additional exposures from the towers in both remote and human populated areas. Well-intentioned conservation organizations may be relying on a degree of safety that does not exist. Plus, the growing popularity of Motus networks at conservation sites, which are often staffed by volunteers, raises questions of training adequacy. The capture, tagging, and release of wildlife species require a high degree of proficiency to keep species safe. How standardized is that training from site-to-site? It is possible that the combination of all these factors to individual tagged species, plus added RFR ambient levels from the infrastructure, could be a contributory factor to species diminishment. This needs to be more broadly and thoroughly studied with an eye toward decreasing such sites, not creating a seamless network along entire migratory routes.

In looking at the maps of where the Motus network is the most built out (95), those are areas where species diminishment is the most pronounced, which is not to infer automatic cause/effect but rather to call for better study and integration of all variables, including EMFs, into the many causes of species diminishment today. There is no post-construction or tagging surveillance being conducted by the federal government or the wildlife community, and the branch of science—the bioelectromagnetics community—that knows the most about this needs to be included.

While, to date, no radiation standards for nonhuman species have been developed, let alone implemented, by the U.S. Federal Communications Commission, the U.S. Environmental Protection Agency, or any international standards setting entity, the concerns continue to escalate, especially as the world’s wildlife continues to decline due to the ongoing sixth massive global species extinction event—the so-called Anthropocene (“Age of Man”) (97–99). What role EMF is playing as a part of this loss of species is of great potential concern, given nonhuman species’ unique physiology that evolved over millennia in a sensitive harmony/relationship with the Earth’s natural geomagnetic fields (3–6) upon which they depend for all life’s activities, and the fact that this is the fastest rising environmental exposure today. It is logical to assume that artificial EMFs are capable of affecting species with distinctive magnetoreception mechanisms and physiologies far more sensitive than humans, given the unusual signaling characteristics, odd wave forms, phased pulsing patterns, concentration of nonionizing radiation frequencies at the Earth’s surface and in lower atmospheric regions for the first time in evolutionary history, and at transmission intensities unlike anything in nature. It is also logical to assume that our constantly rising EMF ambient levels are capable of ecosystem level effects to myriad species.

Even if effects from animal radio-tagging prove to be small, as telemetry use continues to scale up, such devices will never-the-less be contributing to all the other effects from environmental EMF that wildlife and domestic pets encounter today—e.g., from cellular communications, wi-fi, emergency broadcast, TV/radio towers, other forms of microwave communications, smart meters/technologies, transmission power lines/substations, satellites, and more. Cumulative effects need to be addressed. This is, of course, in addition to the negative cumulative impacts from climate change, invasive species, habitat loss and degradation, pesticides, contamination and oil spills, poisoning, and others. Wildlife are facing growing threats, akin to “death by a thousand cuts.”

Wildlife professionals have a moral obligation and responsibility to base their research and field studies on sound science, and that should include consideration of the impacts from EMF. We need to seriously question if the continued use of radio tracking devices, gear, and technologies are worth their impacts on the species whose populations we supposedly are trying to protect and maintain. This is more than just an ethical question; we need to know if the use of tracking gear is doing more harm to target species than the benefits accrued from the use of these technologies, especially when used on/in endangered populations where every subject animal counts enormously and may be critical to the species’ very survival. That question—which has yet to be answered—is one that the authors of this paper strongly recommend be pursued.

## Author contributions

AM: Writing – original draft, Writing – review & editing. BBL: Writing – review & editing. HL: Writing – review & editing.
